# Chronic Pain and Posttraumatic Stress Among Patients in Substance Use Treatment: Protocol for NOR-APT, a Longitudinal Cohort Study

**DOI:** 10.2196/67663

**Published:** 2025-12-24

**Authors:** Ingeborg Skjærvø, Anne Marciuch, Linn Wergeland Digranes, Lena M Follerås, Jon Mordal, Kristin Klemmetsby Solli, Eli Kristine F Abel, Kim Amundsen, Karina Egeland, Britt Karin Haugen, Bjørn Holmøy, Jan Gunnar Skoftedalen, Bente M Weimand, Lars Tanum

**Affiliations:** 1 Department of Addiction (ARA) Mental Health and Addiction Services Akershus University Hospital Lørenskog Norway; 2 Norwegian Centre for Violence and Traumatic Stress Studies Oslo Norway; 3 Division of Mental Health and Addiction Sykehuset i Vestfold Tønsberg Norway; 4 Department of Research and Development Mental Health and Addiction Services Akershus University Hospital Lørenskog Norway; 5 Faculty of Health, Social and Welfare Studies University of South-Eastern Norway Drammen Norway; 6 Section for Outpatient Addiction Treatment Oslo University Hospital Oslo Norway; 7 Division of Mental Health and Addiction Telemark Hospital Trust Skien Norway; 8 Norwegian Confederation of Addiction Oslo Norway

**Keywords:** addiction, dependence, substance use, drug use, chronic pain, posttraumatic stress disorder, trauma, violence, injury, somatic

## Abstract

**Background:**

Chronic pain conditions and posttraumatic stress disorder (PTSD) are highly prevalent among patients with substance use disorders (SUDs). Both can impact outcomes of SUD treatment and quality of life. There is a need for a large-scale study on the overlap of and interactions between SUD, chronic pain, and PTSD.

**Objective:**

The Norwegian Addiction, Pain and Trauma study (NOR-APT) is the first longitudinal study to describe how substance use and outcomes of SUD treatment are impacted by (1) chronic pain and pain characteristics and (2) interactions between comorbid chronic pain and PTSD.

**Methods:**

Self-reported questionnaire data were collected from patients in all types of SUD treatment at four hospital sites in Norway. The questionnaire data on substance use, pain, and posttraumatic stress symptoms will be combined with retrospective and prospective longitudinal data from high-quality demographic and health registries. The registry data cover, for example, treatment episodes, diagnoses, and prescribed medications and socioeconomic variables, with a follow-up period of altogether 20 years (approximately 2008-2029).

**Results:**

Questionnaire data were collected during March 2021-June 2024. Altogether 1890 patients were approached, and 1645 (87%) questionnaires were completed. The estimated final sample size pending data cleaning (eg, removal of duplicates and validation of consent forms) is 1400-1500. Linkage of registry data for approximately 1000 with valid ID numbers and consent is planned for 2026 (retrospective) and 2029/2030 (prospective). At least 10 publications are planned in the period 2025-2028, based on funding received from the Foundation Dam, the Norwegian Research Council, Akershus University Hospital, and Oslo University Hospital. We plan to apply for further funding related to the use of the prospective registry data.

**Conclusions:**

Results from the NOR-APT study will contribute to a better understanding of SUD, chronic pain and PTSD comorbidities, their interactions, trajectories and impact on SUD treatment outcomes, and subjective quality of life. Results can also contribute knowledge toward the development and assessment of treatment interventions that can improve SUD treatment outcomes.

**Trial Registration:**

ClinicalTrials.gov NCT04908410; https://clinicaltrials.gov/study/NCT04908410

**International Registered Report Identifier (IRRID):**

DERR1-10.2196/67663

## Introduction

### Background

Substance dependence, chronic pain, and posttraumatic stress disorder (PTSD) are global burdens, and all three have been labeled as important public health concerns [1,2]. Recent reports show that the level of substance use [2], the level of chronic pain [1], and the level of trauma that can lead to PTSD [3-5] are stable or on the rise, both in Norway and internationally. Additionally, substance use, chronic pain, and PTSD often interact or overlap. Recent evidence points toward chronic pain, also called persistent pain, as a central comorbidity for patients with substance use disorders (SUDs) [6-16]. Chronic pain is a condition with serious detrimental effects on function, quality of life, family and social life, as well as mental health [17-19]. Comorbid SUD and chronic pain complicate the patients’ condition [20] and may contribute to both the development and maintenance of a substance dependence [21-25].

There are few high-quality studies that describe chronic pain among patients in SUD treatment, and none describe how comorbid pain affects SUD treatment outcomes, function, and quality of life. Previous studies have had limitations, such as focus on a small subgroup of patients (eg, patients who have developed a dependence on their pain medication or patients receiving a specific medication), a small sample size, or limited data on pain characteristics. Although limited, existing literature supports a high prevalence of chronic pain among populations with opioid dependence (37%-57%) [6-8,11-15] and alcohol dependence (54%) [9]. Studies of pain in cannabis- and stimulant-dependent populations are almost nonexistent, despite the documented pain-relieving effects of the two substances [26,27] and evidence of a relationship between cannabis and chronic pain in other populations [28,29]. Stimulant use has been identified as a potential risk factor for chronic pain [30], and a link between pain and benzodiazepine use has also been demonstrated [31]. The conclusions of existing studies demonstrate a need for longitudinal studies with pain as a primary focus, and with sufficient sample size to examine sex differences [16,29,32].

In general populations, there is evidence that chronic pain co-occurs and interacts with PTSD [33-35]. Despite the high rates of chronic pain, trauma, and PTSD among patients with SUDs [36-40], the overlap and interactions between chronic pain and PTSD have not been much investigated among patients in SUD treatment, who are often excluded from studies on PTSD [41]. There are only two small studies (n=133 and n=170) on the overlap between chronic pain and PTSD in populations with SUDs; only patients with opioid dependence were included, and they could not draw firm conclusions due to low statistical power. The studies support more trauma and higher PTSD symptom levels among patients with comorbid chronic pain [42,43] and conclude that there is a need to better understand the trajectory of development of comorbid SUD, chronic pain, and PTSD and better understand how comorbid chronic pain and PTSD affect SUD treatment outcomes [42,44].

Increased knowledge of interactions between these three conditions may be of great importance to improve and tailor SUD treatment, as comorbid SUD, chronic pain, and PTSD can hinder positive outcomes of SUD treatment [45,46].

The Norwegian Addiction, Pain and Trauma (NOR-APT) study is the first to fill the need for a large, longitudinal study with a primary focus on pain and PTSD among patients in SUD treatment. By combining self-reported questionnaire data with retrospective and prospective data from health registries, the study has a follow-up period of patients of up to 15 years retrospectively and 5 years prospectively. The study will provide knowledge of how chronic pain and PTSD impact SUD treatment outcomes and patients’ quality of life. The planned sample size allows perspectives on sex and different types of substance use.

### Primary Aims and Research Questions

The overarching aim of the NOR-APT study is to generate new knowledge on the relationships between substance use and SUDs, chronic pain, and mental health (with a focus on PTSD), which in turn can contribute toward updated guidelines, individualized treatment, and development and evaluation of new treatments. Aims and research questions are further detailed in Table 1.

**Table 1:** Aims, research questions (RQs), and data sources.

Aims and RQs					Data sources
**Chronic pain in patients with different types of substance use**					
	**Aim 1:** **estimate and describe the need for pain treatment among people in SUD^a^ treatment, and how comorbid pain impacts SUD treatment outcomes**				
		**RQ1: What is the chronic pain prevalence, pain intensity, and interference for patients ...**			Dataset 1^b^
			a. ... with opioid use?		
			b. ... with alcohol use?		
			c. ... with stimulant-, cannabis-, and other substance use?		
		RQ2: What is the cause and location of the reported pain?			Dataset 1
		RQ3: Is there a relationship between substance use or pain characteristics and chronic pain severity?			Dataset 1
		RQ4: Is there a relationship between chronic pain characteristics and factors related to opioid agonist treatment (eg, medication type, dose, and time in agonist treatment)?			Dataset 1
		RQ5: To what degree have patients with self-reported chronic pain been diagnosed and received treatment in the primary or specialist health care services?			Dataset 2^c^
		**RQ6: Are there differences between those who have received pain treatment and not when it comes to ...**			Dataset 2
			a. ... substance use characteristics?		
			b. ... outcomes related to substance use, pain, or quality of life?		
**The overlap between chronic pain and PTSD^d^ symptoms**					
	**Aim 2:** **explore the overlap between chronic pain and PTSD and received treatment for PTSD**				
		RQ7: What is the prevalence of overlapping chronic pain and PTSD?			Dataset 1
		RQ8: Does pain characteristics (pain intensity, cause, location, and interference from pain on functioning) differ for patients with and without PTSD?			Dataset 1
		RQ9: Are there differences in pain characteristics according to the type of traumatizing event and level of posttraumatic symptoms?			Dataset 1
		RQ10: To what degree have patients with self-reported high levels of PTSD symptoms been diagnosed with and received treatment for PTSD?			Dataset 2
		RQ11: Are there differences in substance use and pain outcomes for patients who have received PTSD treatment compared to those who have not?			Dataset 2
	**Aim 3:** **explore the trajectory for the development of comorbid SUD, chronic pain, and PTSD**				
		RQ12: How is the onset of substance use related to the onset of chronic pain and PTSD?			Dataset 1
		RQ13: Can distinct trajectories for the development of the three comorbidities be identified and described in relation to SUD treatment outcomes?			Dataset 1
**The impact of chronic pain on quality of life**					
	**Aim 4:** **explore the relationship between chronic pain characteristics and quality of life**				
		RQ14: Does quality of life differ between patients with and without chronic pain?			Dataset 1
		**RQ15:** **Are there any associations between quality of life and ...**			Dataset 1
			a. ... pain characteristics, including pain intensity, duration, location, or cause?		
			b. ... substance use (type of substances, frequency of use, age first used, and intravenous use)?		
			c. ... type of medication received (methadone, buprenorphine, or buprenorphine-naloxone), dosage, and duration of opioid agonist treatment? (Patients in opioid agonist treatment only)		
**Mental health comorbidities among patients with chronic pain**					
	**Aim 5:** **explore the prevalence of mental health comorbidities and associations with chronic pain**				
		RQ16: What is the prevalence of psychiatric diagnoses other than PTSD (eg, depression, anxiety, personality disorders, attention-deficit/hyperactivity disorder) among patients with and without chronic pain?			Dataset 2
		RQ17: What was the trajectory for first experiencing chronic pain and being diagnosed with any psychiatric diagnosis?			Dataset 2
**Predictors of future outcomes**					
	**Aim 6:** **explore whether self-reported factors at baseline impact outcomes in the 5-year follow-up period**				
		**RQ18:** **Is self-reported chronic pain and pain characteristics associated with ...**			Dataset 3^e^
			a. ... health-related outcomes in the 5-year follow-up period (eg, use of health services and prescribed medications, diagnoses received, employment status and income, and mortality)?		
			b. ... inclusion and retention in opioid agonist treatment or choice of medication type in opioid agonist treatment?		
		RQ19: Is self-reported PTSD, type of traumatizing event, or level of PTSD symptoms associated with health-related outcomes in the 5-year follow-up period (eg, use of health services and prescribed medications, diagnoses received, employment status and income, and mortality)?			Dataset 3
**Sex differences across all RQs**					
	**Aim 7:** **explore sex differences in characteristics and outcomes**				
		**RQ20:** **Are there sex differences in ...**			Dataset 1-3
			a. ... pain characteristics and the impact of pain on function?		
			b. ... the proportion who have received pain treatment?		
			c. ... interactions between chronic pain and posttraumatic stress symptoms?		
			d. ... the proportion who has received a PTSD diagnosis and treatment?		
			e. ... the proportion who has received any psychiatric diagnosis?		
			f. ... impact of pain on quality of life?		

^a^SUD: substance use disorder.

^b^Dataset 1: cross-sectional questionnaire data.

^c^Dataset 2: retrospective registry data linked with cross-sectional data.

^d^PTSD: posttraumatic stress disorder.

^e^Dataset 3: prospective 5-year follow-up registry data linked with cross-sectional and retrospective registry data.

## Methods

### Design

The NOR-APT study is a longitudinal cohort study. Cross-sectional questionnaire data will be combined with both retrospective and prospective health care and demographic registry data. We will publish from three datasets: self-reported baseline data (dataset 1: cross-sectional), retrospective registry data linked with the cross-sectional data (dataset 2: retrospective cohort), and prospective registry data linked with the cross-sectional and retrospective data (dataset 3: prospective cohort).

**Figure 1:**
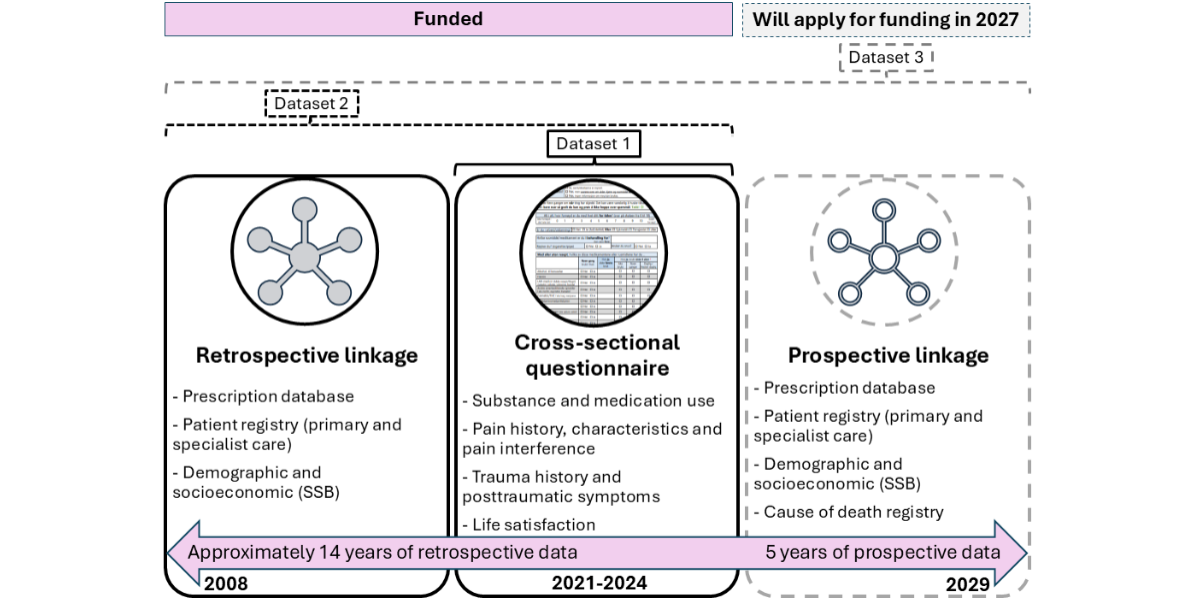
Visualization of study design, datasets, funding, and time frames. SSB: Statistisk Sentralbyrå.

The combination of self-reported questionnaire data with health and demographic registries is an underused study design [47], which provides longitudinal data with minimal strain on the participating clinics and patients. The retrospective health and treatment data will go back to the patient’s first registration in each registry since the registries were established in 2008/2010, giving retrospective data for at least a 14-year period. The prospective data will have a follow-up period of 5 years from the time of completing the questionnaire. Altogether, the data will cover approximately a 20-year period from 2008 to 2029 (Figure 1).

### Setting

In Norway, SUD treatment is available free of charge or with a maximum deductible of up to about US $350 per year. This means data on patients in treatment will be less prone to selection bias related to the private economy compared to countries with different health care systems. Data were collected from inpatient, outpatient, and detoxification clinics at four participating hospitals covering catchment areas with urban, suburban, and rural areas with a total population of over 1,500,000: Akershus University Hospital (AUH), Oslo University Hospital (OUH), Vestfold Hospital Trust, and Telemark Hospital Trust. These four hospital sites together serve about one-fourth of patients receiving SUD treatment in specialist health care in Norway [48].

### Participants and Sample Size Calculations

Participation was open to all patients (age ≥18 years) receiving any SUD treatment from detoxification, inpatient, and outpatient units at the four participating hospitals. Exclusion criteria were a lack of capacity to give informed consent or that physical or mental health concerns made participation too strenuous. Staff recruiting patients for the study were asked to take acute withdrawal, acute intoxication, and serious mental or physical health concerns into account when assessing capacity to give informed consent and to participate.

Based on previous studies, the expected prevalence of chronic pain was 30%-50% [8,9,18]. To achieve a sufficient sample size for detecting a chronic pain prevalence of 30% (α=.05; power=0.80), we needed 384 patients in each substance use subgroup (primarily in treatment for opioids, alcohol, stimulants, cannabis, or benzodiazepines). To detect a prevalence of 50%, 323 patients were necessary. We aimed to include a minimum of 350 patients in each substance use subgroup to provide sufficient power for comparisons between groups (eg, independent samples 2-tailed *t* tests require n=105 in each group) and adjusted regression analyses. Throughout data collection, we adopted recruitment strategies to reach 350 participants within each of the substance use subgroups, focusing recruitment on specific subgroups when necessary. Reaching 350 patients for the opioid and alcohol groups was feasible without targeted measures. Reaching 350 patients who were primarily in treatment for cannabis, stimulants, and benzodiazepines proved challenging, even though we implemented targeted recruitment for these groups to increase the number of patients. However, many patients were in treatment for polysubstance use, and we will be able to take this into account in analyses and can use this to increase sample statistical power (eg, by comparing patients with daily benzodiazepine use to those without while controlling for other substance use, regardless of whether benzodiazepine was named as the primary target in the treatment).

### Procedure and Data Security

Data collection was rolled out in a stepwise manner (AUH: March 23, 2021, to June 27, 2024; OUH: March 14, 2022, to May 21, 2024; Vestfold Hospital Trust: September 13, 2022, to May 22, 2024; and Telemark Hospital Trust: June 2, 2023, to May 2, 2024). The first start of data collection at AUH coincided with societal lockdowns due to the COVID-19 pandemic, which resulted in a slow beginning and contributed to the delayed start-up at the three other hospitals.

The clinics received close follow-up and training from the project group in the start-up phases at each hospital and throughout the data collection period. The training consisted of information on the background for and aims of the study, how to present the study to patients, and how to guide patients in filling out the consent form and questionnaire, with a focus on avoiding recruiter selection bias and missing data. Clinicians were specifically asked to recruit from all patients, not only patients with known pain, or “easy” or better-functioning patients who would not need help completing the questionnaire. Training was provided through in-person or digital staff meetings and discussions. In addition, clinics were provided with two short informational videos and a short manual with tips for how to present the study, common pitfalls in data collection, and additional information about the purpose of the individual questionnaire items. The videos and manual allowed new hires to receive training in recruiting patients for the study, and those who had received training could revisit the information in the available videos and guide.

Clinicians informed the patients about the study and asked if they would participate. The patients completed the written consent form and could choose to provide their unique personal identification number for linkage to health registry databases. Patients who did not want to participate were asked to anonymously provide age, sex, and which substance they consider to be their main problem (these data will be used to assess self-selection bias and generalizability of the findings). Patients who declined providing this information were counted so we can calculate a participation rate.

Clinicians were instructed to help the patients with the questionnaire when needed and to do a quality control of responses to clear up misunderstanding and reduce rates of missing data. Consent forms and questionnaires were prenumbered with matching ID numbers. After completion by the patient, the consent form and questionnaire were kept separately under lock at the participating sites until they were sent securely to the study administration at AUH. Questionnaire data were entered into a database using REDCap (Research Electronic Data Capture) tools hosted at Yale University [49,50] and stored on the AUH secure disc. Unique identifiers linking the patient identities to their data are stored on a server for sensitive research material at AUH. The retrospective and prospective linkage of questionnaire data to registry data will be done by Statistics Norway or Helsedataservice, according to all relevant laws and regulations, and stored on secure servers.

### Measures

#### Cross-Sectional Questionnaire Data

The questionnaire was developed based on literature and input from clinicians in the Pain Resource Group at OUH and the Research Committee at AUH. In total, 10 AUH patients piloted the questionnaire and gave feedback, after which the questionnaire was revised. We used validated questions when available and prioritized short and efficient questions and scales to reduce patient strain.

We collected information on age; sex (male or female); whether employed, a student, on sick leave, on benefits, or retired; and whether they smoked tobacco or used snuff or chewing tobacco (yes or no).

Substance use history and pattern were assessed using adapted questions from section E of the much-used and validated European Addiction Severity Index (EuropASI) interview [51,52]. Patients indicated which substances or medications they have ever used in their lifetime (yes or no), age of first use, and frequency of use in the last 4 weeks (categories collapsed to no use, sometimes, or daily or almost daily). The list of substance categories was revised somewhat to reflect current substance use in the population and the interests of this study. The categories “inhalants” and “more than one substance/medication per day” were removed. The category “other designer drugs, steroids” was removed and replaced with separate categories for “anabolic steroids,” “GHB/GBL” (ie, γ-hydroxybutyrate or γ-butyrolactone), and “khat.” “Alcohol, all use” was dropped, while “alcohol to intoxication” was kept. As the EuropASI is typically conducted as an interview, minor alterations and specifications were added to make it suitable for self-report. The final substance use categories presented in the questionnaire were alcohol to intoxication, heroin, opioid agonist medication (prescribed and illicit, eg, methadone, subutex [ie, proprietary name for buprenorphine], suboxone [ie, proprietary name for buprenorphine-naloxone], and buvidal [ie, proprietary name for long-acting buprenorphine, injected twice weekly]), other opioid-based painkillers (eg, morphine, oxycodone, and tramadol), cannabis or THC (ie, tetrahydrocannabinol, eg, hashish and marijuana), amphetamine or methamphetamine, cocaine, benzodiazepines (eg, valium [ie, proprietary name for diazepam] and sobril [ie, proprietary name for oxazepam]), GHB/GBL, hallucinogens (eg, LSD [ie, lysergic acid diethylamide], mushrooms, mescaline, and MDMA [ie, methylenedioxymethamphetamine]), anabolic steroids, and khat. Patients reported which substance they considered most problematic for them (EuropASI) and also which substance use they were currently receiving treatment for (question devised for this study). Further, patients indicate intravenous substance use in their lifetime and in the last 4 weeks (yes or no). Opioid agonist treatment (OAT) history and relation to pain were assessed by asking whether the first opioid use was to relieve pain (not used opioids, yes, no, or do not know), whether they had ever been enrolled in OAT, the type of medication they were prescribed (both current or previously), medication dose, age when the prescription started, and whether pain had ever influenced choice of medication type in OAT (no, yes, or do not know). If they responded yes, they were asked to elaborate which medication, report their age at the time, and provide a comment to explain.

Pain and pain characteristics included pain in the last 4 weeks (excluding pain from abstinence only; only patients who responded yes were asked to complete the remaining questions about pain), pain duration (<3 months, 3-12 months, 1-3 years, 4-6 years, 7-10 years, and >10 years), and cause of pain (disease, accident or injury, violence, surgery, other, and unknown). Pain location was assessed with a body map from the Norwegian Pain Association [53], where participants could mark up to 25 areas where they feel pain. Based on comments from patients in a previous pain questionnaire [8], we added the option to indicate any general pain in the skin, muscles, joints, or skeleton. We asked whether they had seen a doctor for their pain and what their age was at the time. Further, they were asked whether they received treatment for pain and the type of treatment (this is self-reported data on received treatment available in dataset 1. Additional data on received treatment will be available from the health registries in datasets 2-3). Pain intensity was assessed with four questions from the Brief Pain Inventory Short Form (BPI-SF) [54], a widely used measure validated in Norwegian [55], with responses on a numeric rating scale from 0=no pain to 10=worst imaginable. Pain interference of physical and emotional function was assessed through 7 questions from the BPI-SF [54], asking to what degree pain had influenced daily activity, ability to walk, ability to work or do chores around the house, mood, relationship to others, sleep, and joy of life. Responses were given on a numeric rating scale from 0=not affected to 10=completely interfered.

Exposure to potentially traumatizing events was assessed with a short version of the Stressful Life Events Screening Questionnaire (SLESQ), which lists 15 potentially traumatizing events [56]. Patients indicated which of the 15 events they had been exposed to; however, they were not asked the follow-up questions related to each item in the original questionnaire. Instead, patients were asked what their age was when the first event occurred and their age at the time of the event that caused the most distress. To shorten the questionnaire further, the layout was revised, and text that did not contain information necessary for understanding the questions was removed. This revision was shared with one of the developers of SLESQ to ensure that important concepts were not omitted.

To cover the *DSM-5* (*Diagnostic and Statistical Manual of Mental Disorders* [Fifth Edition]) criteria for PTSD (re-experiencing, avoidance, negative thoughts or emotions, and overactivation), posttraumatic stress symptoms were measured through a 4-item version of the PTSD Checklist for DSM-5 (PCL-5) [57]. Only patients who had indicated at least one potentially traumatizing event were asked to complete the questions on posttraumatic stress symptoms.

Subjective quality of life, specifically life satisfaction, was measured by asking “All in all, how satisfied are you with your life nowadays?” with responses on a scale from 0=not satisfied at all to 10=very satisfied. This 1-item question was developed for UK national questionnaires [58] and has also been used in over 30 countries in the Organisation for Economic Co-operation and Development (OECD) Better Life Index and in Statistics Norway questionnaires on quality of life in the general Norwegian population [59]. Using one general quality of life question has the advantage of measuring subjective quality of life without predefining what should affect an individual’s quality of life. The question was placed at the beginning of the questionnaire to avoid other themes in the questionnaire influencing patients’ mood and response. We added a question in the same format as the BPI-SF [54], asking to what degree pain had influenced how satisfied they were with their lives, with a response scale from 0=not affected to 10=completely interfered.

#### Demographic and Health Registries

The Norwegian registries consist of high-quality data with a complete history of treatment episodes, diagnoses, and prescribed medications [47], as well as socioeconomic variables. Registry data will be linked to self-reported data through personal identification numbers retained by all residents in Norway.

Retrospective registry data (datasets 2-3) will cover information from each patient’s first registration in each registry and will be right-censored at the date on the consent form for completion of the questionnaire. The start date for the existence of the different registries varies from 1953 to 2016 (Table 2). Further, the start dates of different variables within the registries may also vary; for instance, a registry may have been started in 1967, but a specific data point or variable was not included until 2005. Thus, the time periods and left censoring points for analyses on the retrospective data may vary with different research questions and the use of variables. For instance, sociodemographic data are typically available far enough back in time to provide information on childhood conditions for most patients, while the somatic and mental health registries only have data available from 2009.

Prospective registry data (dataset 3) will be available from the date of the participant’s consent, until 5 years following the end of the data collection for the study. As data collection was conducted over several years, some patients will have a follow-up period of 5 years, while others may be closer to 8 years. Depending on the research question, all patients may have prospective data censored at the 5-year point following their completion of the questionnaire (or date of death, or date of emigration from Norway), or all follow-up data will be included in analyses adjusting for patient variation in time-at-risk (with right censoring occurring 5 years after inclusion of the last patient, or date of death, or date of emigration from Norway, if no event of interest has occurred). Registry names and variables for this study are detailed in Table 2.

**Table 2:** List of registries, types of data to be extracted from the registries, and the year each registry was established.

Registry name		Variables or data	Data from
**Primary registry sources**			
	Norwegian Patient Register	Contacts with specialist health care services (somatic, mental health, and substance treatment services) Time of treatments ICD-10^a^ codes for the treatments	2009
	The municipal user and patient register (KPR^b^)	Contacts with the primary health care services (primary physicians, dentists, municipal mental health services, and emergency rooms) Time of services Reimbursement codes linked to ICD-10 codes	2016
	Prescription Database (NorPD^c^)	All prescriptions dispensed from pharmacies or institutions Date Medication type and dosage Profession of the prescriber	2004
	Statistics Norway (SSB^d^)	Sociodemographic variables at the individual and household level Level of education Living conditions Family situation (household, spouse or partner, and children) Birth country Employment status Income Benefits Sick leave	1967
	Cause of Death registry	Date of death ICD-10 codes related to the cause of death	5-year follow-up data
**Secondary registry sources^e^**			
	National Quality Registry for Pain Management (SmerteReg)	Contains information on treatment, pain intensity, and function for those participants who have been referred to teams for acute pain treatment at Norwegian hospitals	2014
	Cancer registry	Type of cancer Time of diagnosis and treatment Types of treatment	1953
	National Trauma Registry	Treatment for serious physical injuries by trauma teams at hospitals Time of injury Severity and type of injury Type of accident (eg, traffic, violence, work, and self-harm) Level of treatment	2014

aICD-10: International Statistical Classification of Diseases, Tenth Revision.

^b^KPD: Kommunalt Pasientregister.

^c^NorPD: Norwegian Prescription Database.

^d^SSB: Statistisk Sentralbyrå.

^e^We have consent from participants to retrieve data from these registries; however, as entry into the registries is dependent on specific conditions or events, we do not know whether there will be a sufficient number of participants present in these secondary registries to warrant analyses of data.

### Datasets, Outcomes, and Analyses

#### Overview

Research questions and a preliminary analysis strategy are part of the preregistration on ClinicalTrials.gov (NCT04908410). This is an exploratory study; thus, there are no preregistered hypotheses.

The study involves two types of data (self-reported baseline cross-sectional data and longitudinal registry data), which will be combined into three different datasets with three different research designs (datasets 1-3).

#### Dataset 1: Cross-Sectional (Self-Reported Baseline Data)

The primary outcomes will be (1) self-reported mild, moderate, or severe chronic pain (duration of at least 3 months; *ICD-10* [*International Statistical Classification of Diseases, Tenth Revision*]) and (2) self-reported PTSD symptoms. Additionally, secondary outcomes will be related to pain (pain etiology and characteristics; pain treatment; impact of pain on substance use, opioid use, and choice of type of opioid agonist in OAT; and substances or medications that improve or worsen pain), trauma (types of potentially traumatizing events and age of the first event and the event that causes the most distress), and quality of life (current life satisfaction and impact of pain on life satisfaction).

The primary statistical models will be regression analyses with chronic pain and PTSD symptoms as outcomes, adjusting for relevant background, substance use, and pain characteristics. Secondary or explorative analyses include regression models with quality of life as outcome and chronic pain or PTSD comorbidity as primary predictors. Further, we will explore these models and the primary models within different subgroups of substance use (eg, alcohol only, alcohol combined with opiates, and stimulant use). We will use appropriate comparison tests (eg, 2-tailed *t* tests and chi-square tests) to compare patients with and without chronic pain, both in the total sample and substance use subgroups separately, and to compare patients with chronic pain and PTSD overlap to those who have neither or only one of the conditions.

#### Dataset 2: Retrospective Cohort (Retrospective Registry Data Linked With Cross-Sectional Data)

The primary outcomes will be (1) the proportion of patients who have received pain treatment (treatment for a pain-related *ICD-10* diagnosis) and (2) self-reported substance use at baseline.

Secondary outcomes include outcomes related to pain (pain intensity, pain interference, and impact of pain on substance use), the proportion of patients who are registered with a diagnosis or treatment for PTSD or other psychiatric disorders, as well as self-reported PTSD symptoms, quality of life, and dosage of OAT medication (when relevant).

The primary model will be a regression analysis with substance use as the outcome, with received pain treatment as the primary predictor (exposure), adjusting for other relevant sociodemographic and pain variables. Further, secondary or explorative analysis will include regression models with pain intensity and pain interference as outcomes, with received pain treatment as the primary predictor (exposure), and regression models with received PTSD treatment as the primary predictor (exposure), and PTSD symptoms, substance use, and pain intensity as outcomes. We will also use appropriate comparison tests (eg, 2-tailed *t* tests and ANOVA) between patients who have received pain treatment and PTSD treatment and those who have not.

#### Dataset 3: Prospective Cohort (Prospective Registry Data Linked With Cross-Sectional and Retrospective Data)

The primary outcomes will be time to first treatment for substance use and the number of treatment episodes following the index treatment (during which the questionnaire was completed). Secondary outcomes will be general health service use, prescribed medications, diagnoses and treatment related to pain and mental health, employment and income, housing, retention in OAT (when relevant), and mortality.

The primary model will be time-to-event analyses that take differing time-at-risk into consideration (eg, Cox proportional hazards model) to assess associations between chronic pain as an exposure and risk of new treatment episodes for substance use, controlling for other baseline variables. Secondary or explorative analyses will include regression analyses (such as logistic or linear regression) to investigate associations between chronic pain or PTSD and secondary outcomes, adjusting for relevant sociodemographic variables.

#### All Datasets

We will use network analyses (mixed graphical models) to explore which variables (demographics, substance use, pain, and mental health) are most strongly related to positive SUD treatment outcomes [60] and how the variables relate to each other, as well as explore the centrality and bridge centrality of different variables [61,62].

When sample size allows, comparisons between male and female populations will be made using appropriate statistical tests, and sex will at the very least be adjusted for in statistical models.

### Ethical Considerations

The study was approved by the regional ethics committee south-east (REK/173249) and by the privacy office of the hospital sites. The study is registered on ClinicalTrials.gov (NCT04908410). Several measures were taken to ensure that participation was not a burden for the patients: (1) participation was voluntary, and provision of treatment was not contingent upon it. Written and informed consent was obtained. Patients could choose to only fill out the questionnaire or to also provide their personal identification number for linkage to health registries. (2) Ability to give informed consent was assessed, and patients were able to withdraw at a later stage by contacting the local or national principal investigator. (3) If the content of the questionnaire were to trigger negative feelings, clinicians were available to support the patients. (4) We focused on making the questionnaire short and simple to reduce the burden and time spent by the patients. (5) If the patient wanted to, they could discuss their responses to the questions in the questionnaire with their treatment provider, and potentially, new information disclosed in the questionnaire could directly benefit the patient. Patients were not compensated for their participation.

### Training of Clinicians Who Recruited Patients

Clinicians were informed about the purpose of the study and trained in how to present participation and how to help patients complete the questionnaire to ensure high-quality data. Potential negative reactions patients might have to the themes in the questionnaire were also discussed with the clinicians, as well as the potential benefits for the patients if they wished information from the questionnaire to be used in their treatment.

### Risk for the Patients and the Project

**Figure 2:**
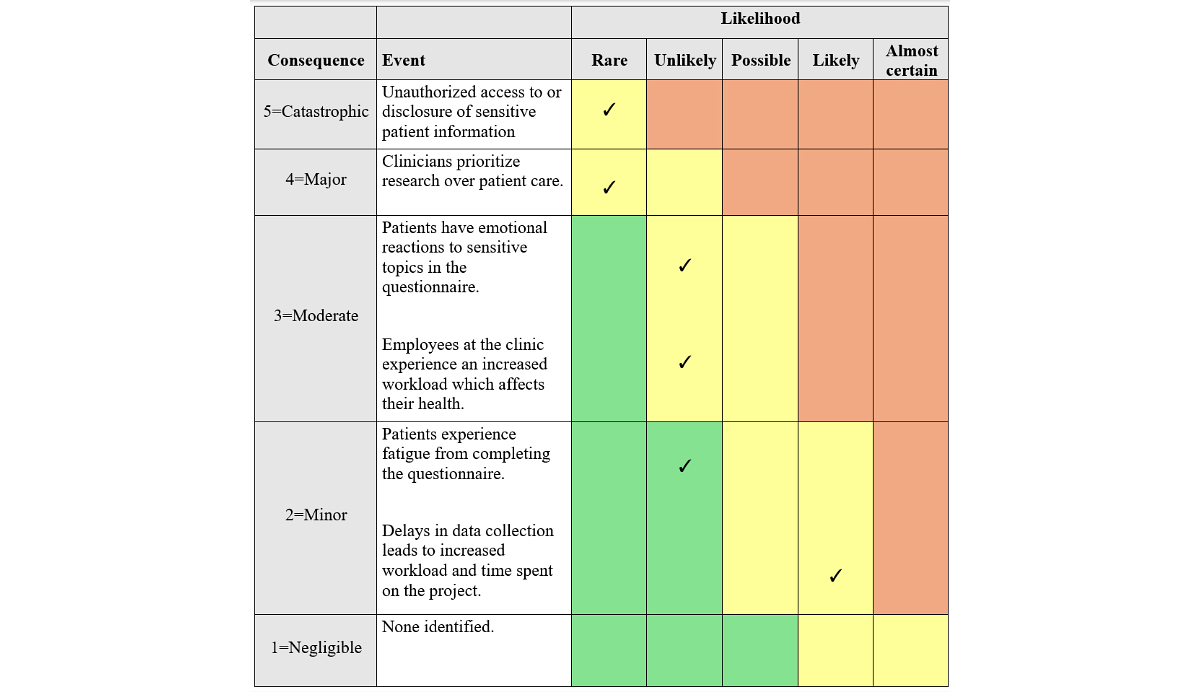
Risk analysis of adverse events in the project.

We considered that there was a very limited risk or strain for patients as a result of participating in the research project. There were no disadvantages for patients if they chose not to participate. Events that could have serious consequences but were considered unlikely to occur given our study procedures (Figure 2) include identifiable sensitive patient data coming into the hands of someone outside the research group or clinicians prioritizing study participation over treatment. Moderately serious events that we assumed could happen in a few cases were patients having emotional reactions to some of the questions in the questionnaire or becoming fatigued. In these cases, the clinicians were instructed to follow the procedures of the clinics and take care of the patients. The project group has received reports of a few instances where patients reacted with great emotional upset to questions related to traumatic experiences; the clinics followed up with the patients and ensured their safety.

## Results

### Data Collection and Funding

The questionnaire data were collected between March 23, 2021, and June 27, 2024. Altogether 1890 patients were asked to participate, of which, 1645 (87%) completed the questionnaire. As of June 2025, responses have been entered into a database and are undergoing data cleaning (eg, removal of duplicates and validation of consent forms). Final sample size with unique participations and valid consent has not been determined, but it is estimated to be between 1400 and 1500.

Of the 1648 patients who completed the questionnaire, 1382 were also asked if they would provide their personal ID for linkage with registry data. The number of unique participants with valid consent and ID numbers for linkage to the registries is estimated to be approximately 1000-1100, which is about 76% of the 1382 who were asked for their personal ID, with the exact number pending validation of consent forms. Linkage with retrospective and prospective registry data is planned for 2025/2026 and 2029/2030, respectively.

The NOR-APT study is funded by the Foundation Dam (2024-2026), the Norwegian Research Council (2024-2027), AUH (2025), and OUH (2023). We plan to apply for further funding of the prospective registry data (dataset 3). The current funding covers the principal investigator, a 3-year postdoctoral position, a 3-year PhD position, and project costs. In total, 10 publications are planned in the period 2025-2028, based on the existing funding.

### Study Progress

Data collection was completed in June 2024. We have entered all data into a database using REDCap [49,50]. Per June 2025 (the time of submitting this protocol), we are working on cleaning and finalizing the dataset, including removing duplicate participations and participations with unclear or nonvalid written consent.

## Discussion

### Expected Impact

Findings from the NOR-APT study will challenge current practice and knowledge in several ways. NOR-APT results will contribute to a foundation for the development and assessment of treatment interventions that can improve SUD treatment outcomes. Further, increased knowledge of the trajectories of developing comorbid substance use, chronic pain, and PTSD can also contribute to the development of early or preventive interventions to reduce the likelihood of developing comorbidities when one of the conditions is present. The study results can improve current knowledge and practice, both in the short term, through implementation of standardized screening to uncover treatment needs related to pain and trauma or recruitment of staff with pain and trauma competence, and in the long term, by stimulating interest in and studies on interventions for comorbid SUD, chronic pain, and PTSD.

Results are generalizable to SUD treatment in international contexts and may also be relevant for some related fields; for instance, findings among patients who are opioid-dependent can contribute to the ongoing discussion of opioid treatment of pain in pain clinics.

As a clinic-driven initiative, with a public hospital as the project owner, we are close to patients, clinicians, and clinic leaders. The NOR-APT study addresses major knowledge gaps related to comorbid chronic pain and PTSD among patients in SUD treatment. Currently, chronic pain and PTSD are typically not routinely screened for during SUD treatment; we assume the results from the study will show the importance of identifying these comorbidities and the importance of having guidelines for how these comorbidities can be included as targets in SUD treatment.

Further, awareness of the high prevalence and impact of comorbid chronic pain and PTSD can cause treatment clinics to recruit employees with expertise within chronic pain and trauma or provide broader training to clinicians to increase their confidence and skills in treating patients with these comorbidities. This can improve outcomes for patients and reduce costs for the clinics in the long-term, through more efficient treatment. Clinicians report that the assessments included in the study have already uncovered pain conditions and posttraumatic stress symptoms in patients that were previously not known to the clinics, and this is shedding new light on their substance use and treatment needs.

Finally, we are convinced that the results will contribute to the willingness to continue work toward evidence-based practices, through intervention studies for chronic pain management and PTSD among patients in SUD treatment.

### Strengths

The NOR-APT study is to our knowledge the largest longitudinal study to investigate comorbid chronic pain and PTSD among patients in SUD treatment. Participation being open to patients with all types of substance use allows comparisons between patients with different substance use patterns, in the same catchment areas, using the same measures. The primary goal was to assess pain and PTSD; thus, the questionnaire includes a broad assessment of pain and trauma-related factors. The linkage of complete records from demographic and health registries with questionnaire data allows demographics and treatment history from registries to be seen in relation to the patients’ self-reported pain characteristics, substance use patterns, and quality of life. It further allows the questionnaire data to be used in relation to other mental health disorders than PTSD, as information on all diagnoses the patients have received will be available.

### Limitations

The questionnaire provides self-reported data, where there is a risk of self-report bias (such as memory bias and social desirability bias). It is also possible that there could be some self-selection bias where patients who found the themes most relevant (eg, patients with pain) may have agreed to participate, while patients who did not have pain saw participation as less relevant and declined. This could inflate pain prevalence estimates.

Although clinicians were asked to inform all patients about the study, regardless of any known pain or trauma, it is possible that clinicians may have had a bias toward presenting study participation to patients they believed would be more likely to say yes (eg, better functioning patients). Clinicians who had missed trainings and not seen the training materials could have believed the study was only relevant for patients with pain and thus selectively recruited patients with known pain. We explained and reminded clinicians of these potential biases throughout data collection and repeated that all patients, regardless of functioning and pain, should be included. Therefore, we assume that most cases of patients not being asked to participate would be related to the clinic’s capacity and not factors related to the patient. At the same time, we are not naïve to the likely presence of some bias in our sample, for example, related to a preference for recruiting “easier patients” (which could reduce representativity for all patients in treatment or reduce pain estimates if these patients have more pain) or where some clinicians through misunderstanding recruited only patients with known pain (which would inflate our estimates of pain). We will compare our results to other studies and discuss these potential biases in our publications.

As the questionnaire was only available in Norwegian, patients who did not speak Norwegian could participate if clinicians helped them by translating the questions for them; thus, there is likely a selection bias where patients who did not speak Norwegian participated to a lesser degree. However, though no official statistics have been published, the proportion of patients in substance use treatment in Norway who do not speak Norwegian is fairly low, and language barriers were not reported as a challenge by the recruiting clinics. To assess the representability of our sample compared to the entire population of patients in treatment for substance use at the participating hospitals, and Norway overall, we will attempt to get the whole population data on immigration status from Statistics Norway. If the participation of patients with an immigration background is skewed, we will conduct weighted analyses.

Further, we can explore potential biases in participation related to age, sex, and which substance patients considered the most problematic for them, as we collected information on this from patients who declined participation. With self-reported data, missing or unclear responses can also be an issue, although clinicians were instructed to check the questionnaires and ask patients about any unclear responses.

All patients were receiving assessment or treatment from clinics for substance use and dependence treatment in the specialist health services. However, in the cross-sectional questionnaire, we did not ask patients to specify their SUD-related diagnoses, and we asked only about the use of substances, including medications, which substance they were receiving treatment related to, and which substance they perceived as most problematic. Thus, the questionnaire data only contain proxies for SUD. In the registry data, however, all *ICD-10* codes for SUD, mental health disorders, and somatic diseases will be available.

Concerning registry data, there is little risk of bias, as these data are not self-reported, and records for all patients will be complete or nearly complete, with little missing data. The limitation of registry data is that it only has information from official records; thus, undiagnosed conditions or other relevant events that are not part of the registers will not be available. Although the patients included in this study are in contact with service providers, it is possible that patients in SUD treatment are less likely to seek, be assessed for, and receive health care services related to other conditions, leading to underreporting of health problems from registries. The likely prevalence of PTSD and chronic pain can be estimated from the self-reported questionnaire data; however, this will not be possible for other mental health disorders or somatic conditions.

## Appendix

Multimedia Appendix 1 Peer-review reports by the Research Council of Norway.

## Abbreviations

AUH: Akershus University Hospital
BPI-SF: Brief Pain Inventory Short Form
DSM-5: Diagnostic and Statistical Manual of Mental Disorders, Fifth Edition
EuropASI: European Addiction Severity Index
GBL: γ-butyrolactone
GHB: γ-hydroxybutyrate
ICD-10: International Classification of Diseases, Tenth Revision
LSD: lysergic acid diethylamide
MDMA: methylenedioxymethamphetamine
NOR-APT: Norwegian Addiction, Pain and Trauma
OAT: opioid agonist treatment
OECD: Organisation for Economic Co-operation and Development
OUH: Oslo University Hospital
PCL-5: PTSD Checklist for DSM-5
PTSD: posttraumatic stress disorder
REDCap: Research Electronic Data Capture
SLESQ: Stressful Life Events Screening Questionnaire
SUD: substance use disorder
THC: tetrahydrocannabinol

## Data Availability

The datasets analyzed during this study are not publicly available, as there is no ethics approval or consent from participants to make the data public. Questions regarding the dataset can be directed to the corresponding author.

## References

[1] Zhu M, Zhang J, Liang D, Qiu J, Fu Y, Zeng Z, Han J, Zheng J, Lin L (2024). Global and regional trends and projections of chronic pain from 1990 to 2035: analyses based on global burden of diseases study 2019. Br J Pain.

[2] An J, Wang Q, Bai Z, Du X, Yu D, Mo X (2025). Global burden and trend of substance use disorders, self-harm, and interpersonal violence from 1990 to 2021, with projection to 2040. BMC Public Health.

[3] Dale MTG, Aakvaag HF, Strøm IF, Augusti E-M, Skauge AD (2023). Omfang av vold og overgrep i den norske befolkningen. NKVTS Rapport.

[4] Kessler RC, Aguilar-Gaxiola S, Alonso J, Benjet C, Bromet EJ, Cardoso G, Degenhardt L, de Girolamo G, Dinolova RV, Ferry F, Florescu S, Gureje O, Haro JM, Huang Y, Karam EG, Kawakami N, Lee S, Lepine J, Levinson D, Navarro-Mateu F, Pennell B, Piazza M, Posada-Villa J, Scott KM, Stein DJ, Ten Have M, Torres Y, Viana MC, Petukhova MV, Sampson NA, Zaslavsky AM, Koenen KC (2017). Trauma and PTSD in the WHO World Mental Health surveys. Eur J Psychotraumatol.

[5] Benjet C, Bromet E, Karam EG, Kessler RC, McLaughlin KA, Ruscio AM, Shahly V, Stein DJ, Petukhova M, Hill E, Alonso J, Atwoli L, Bunting B, Bruffaerts R, Caldas-de-Almeida JM, de Girolamo G, Florescu S, Gureje O, Huang Y, Lepine JP, Kawakami N, Kovess-Masfety V, Medina-Mora ME, Navarro-Mateu F, Piazza M, Posada-Villa J, Scott KM, Shalev A, Slade T, ten Have M, Torres Y, Viana MC, Zarkov Z, Koenen KC (2016). The epidemiology of traumatic event exposure worldwide: results from the World Mental Health Survey Consortium. Psychol Med.

[6] Barry DT, Savant JD, Beitel M, Cutter CJ, Moore BA, Schottenfeld RS, Fiellin DA (2013). Pain and associated substance use among opioid dependent individuals seeking office-based treatment with buprenorphine-naloxone: a needs assessment study. Am J Addict.

[7] Higgins C, Smith BH, Matthews K (2020). Comparison of psychiatric comorbidity in treatment-seeking, opioid-dependent patients with versus without chronic pain. Addiction.

[8] Latif Z-EH, Skjærvø I, Solli KK, Tanum L (2021). Chronic pain among patients with an opioid use disorder. Am J Addict.

[9] Boissoneault J, Lewis B, Nixon SJ (2019). Characterizing chronic pain and alcohol use trajectory among treatment-seeking alcoholics. Alcohol.

[10] Peles E, Schreiber S, Adelson M (2009). Documented poor sleep among methadone-maintained patients is associated with chronic pain and benzodiazepine abuse, but not with methadone dose. Eur Neuropsychopharmacol.

[11] Peles E, Schreiber S, Gordon J, Adelson M (2005). Significantly higher methadone dose for methadone maintenance treatment (MMT) patients with chronic pain. Pain.

[12] Rosenblum A, Joseph H, Fong C, Kipnis S, Cleland C, Portenoy RK (2003). Prevalence and characteristics of chronic pain among chemically dependent patients in methadone maintenance and residential treatment facilities. JAMA.

[13] Dunn KE, Finan PH, Tompkins DA, Fingerhood M, Strain EC (2015). Characterizing pain and associated coping strategies in methadone and buprenorphine-maintained patients. Drug Alcohol Depend.

[14] Potter JS, Shiffman SJ, Weiss RD (2008). Chronic pain severity in opioid-dependent patients. Am J Drug Alcohol Abuse.

[15] Stein MD, Herman DS, Bailey GL, Straus J, Anderson BJ, Uebelacker LA, Weisberg RB (2015). Chronic pain and depression among primary care patients treated with buprenorphine. J Gen Intern Med.

[16] Alford DP, German JS, Samet JH, Cheng DM, Lloyd-Travaglini CA, Saitz R (2016). Primary care patients with drug use report chronic pain and self-medicate with alcohol and other drugs. J Gen Intern Med.

[17] Dueñas M, Ojeda B, Salazar A, Mico JA, Failde I (2016). A review of chronic pain impact on patients, their social environment and the health care system. J Pain Res.

[18] Breivik H, Collett B, Ventafridda V, Cohen R, Gallacher D (2006). Survey of chronic pain in Europe: prevalence, impact on daily life, and treatment. Eur J Pain.

[19] Hadi MA, McHugh GA, Closs SJ (2019). Impact of chronic pain on patients' quality of life: a comparative mixed-methods study. J Patient Exp.

[20] Chang Y, Compton P (2013). Management of chronic pain with chronic opioid therapy in patients with substance use disorders. Addict Sci Clin Pract.

[21] Larson MJ, Paasche-Orlow M, Cheng DM, Lloyd-Travaglini C, Saitz R, Samet JH (2007). Persistent pain is associated with substance use after detoxification: a prospective cohort analysis. Addiction.

[22] Edwards S, Vendruscolo LF, Gilpin NW, Wojnar M, Witkiewitz K (2020). Alcohol and pain: a translational review of preclinical and clinical findings to inform future treatment strategies. Alcohol Clin Exp Res.

[23] Pud D, Cohen D, Lawental E, Eisenberg E (2006). Opioids and abnormal pain perception: new evidence from a study of chronic opioid addicts and healthy subjects. Drug Alcohol Depend.

[24] Apkarian AV, Neugebauer V, Koob G, Edwards S, Levine JD, Ferrari L, Egli M, Regunathan S (2013). Neural mechanisms of pain and alcohol dependence. Pharmacol Biochem Behav.

[25] Egli M, Koob GF, Edwards S (2012). Alcohol dependence as a chronic pain disorder. Neurosci Biobehav Rev.

[26] Chen AL, Chen TJ, Waite RL, Reinking J, Tung HL, Rhoades P, Downs BW, Braverman E, Braverman D, Kerner M, Blum SH, DiNubile N, Smith D, Oscar-Berman M, Prihoda TJ, Floyd JB, O'Brien D, Liu H, Blum K (2009). Hypothesizing that brain reward circuitry genes are genetic antecedents of pain sensitivity and critical diagnostic and pharmacogenomic treatment targets for chronic pain conditions. Med Hypotheses.

[27] Hill KP (2015). Medical marijuana for treatment of chronic pain and other medical and psychiatric problems: a clinical review. JAMA.

[28] Manchikanti L, Damron KS, Beyer CD, Pampati V (2003). A comparative evaluation of illicit drug use in patients with or without controlled substance abuse in interventional pain management. Pain Physician.

[29] Zvolensky M, Cougle J, Bonn-Miller MO, Norberg M, Johnson K, Kosiba J, Asmundson GJG (2011). Chronic pain and marijuana use among a nationally representative sample of adults. Am J Addict.

[30] Tiet QQ, Moos RH (2021). Strong associations among PTSD, pain, and alcohol and drug use disorders in VA primary care patients. Drug Alcohol Depend.

[31] Lape EC, Powers JM, Hooker JE, Edwards RR, Ditre JW (2023). Benzodiazepine use and dependence in relation to chronic pain intensity and pain catastrophizing. J Pain.

[32] Witkiewitz K, Vowles KE (2018). Alcohol and opioid use, co-use, and chronic pain in the context of the opioid epidemic: a critical review. Alcohol Clin Exp Res.

[33] Sharp TJ, Harvey AG (2001). Chronic pain and posttraumatic stress disorder: mutual maintenance?. Clin Psychol Rev.

[34] Asmundson GJ, Coons MJ, Taylor S, Katz J (2002). PTSD and the experience of pain: research and clinical implications of shared vulnerability and mutual maintenance models. Can J Psychiatry.

[35] Beck JG, Clapp JD (2011). A different kind of co-morbidity: understanding posttraumatic stress disorder and chronic pain. Psychol Trauma.

[36] Zhang S, Lin X, Liu J, Pan Y, Zeng X, Chen F, Wu J (2020). Prevalence of childhood trauma measured by the short form of the Childhood Trauma Questionnaire in people with substance use disorder: a meta-analysis. Psychiatry Res.

[37] Keyser-Marcus L, Alvanzo A, Rieckmann T, Thacker L, Sepulveda A, Forcehimes A, Islam LZ, Leisey M, Stitzer M, Svikis DS (2015). Trauma, gender, and mental health symptoms in individuals with substance use disorders. J Interpers Violence.

[38] Driessen M, Schulte S, Luedecke C, Schaefer I, Sutmann F, Ohlmeier M, Kemper U, Koesters G, Chodzinski C, Schneider U, Broese T, Dette C, Havemann-Reinecke U (2008). Trauma and PTSD in patients with alcohol, drug, or dual dependence: a multi-center study. Alcohol Clin Exp Res.

[39] Ford JD, Hawke J, Alessi S, Ledgerwood D, Petry N (2007). Psychological trauma and PTSD symptoms as predictors of substance dependence treatment outcomes. Behav Res Ther.

[40] Degenhardt L, Bharat C, Glantz MD, Bromet EJ, Alonso J, Bruffaerts R, Bunting B, de Girolamo G, de Jonge P, Florescu S, Gureje O, Haro JM, Harris MG, Hinkov H, Karam EG, Karam G, Kovess-Masfety V, Lee S, Makanjuola V, Medina-Mora ME, Navarro-Mateu F, Piazza M, Posada-Villa J, Scott KM, Stein DJ, Tachimori H, Tintle N, Torres Y, Viana MC, Kessler RC (2022). The associations between traumatic experiences and subsequent onset of a substance use disorder: findings from the World Health Organization World Mental Health surveys. Drug Alcohol Depend.

[41] Leeman RF, Hefner K, Frohe T, Murray A, Rosenheck RA, Watts BV, Sofuoglu M (2017). Exclusion of participants based on substance use status: findings from randomized controlled trials of treatments for PTSD. Behav Res Ther.

[42] Barry DT, Cutter CJ, Beitel M, Kerns RD, Liong C, Schottenfeld RS (2016). Psychiatric disorders among patients seeking treatment for co-occurring chronic pain and opioid use disorder. J Clin Psychiatry.

[43] Barry DT, Beitel M, Cutter CJ, Garnet B, Joshi D, Rosenblum A, Schottenfeld RS (2011). Exploring relations among traumatic, posttraumatic, and physical pain experiences in methadone-maintained patients. J Pain.

[44] Ecker AH, Hundt N (2018). Posttraumatic stress disorder in opioid agonist therapy: a review. Psychol Trauma.

[45] Witkiewitz K, Vowles KE, McCallion E, Frohe T, Kirouac M, Maisto SA (2015). Pain as a predictor of heavy drinking and any drinking lapses in the COMBINE study and the UK Alcohol Treatment Trial. Addiction.

[46] Torchalla I, Nosen L, Rostam H, Allen P (2012). Integrated treatment programs for individuals with concurrent substance use disorders and trauma experiences: a systematic review and meta-analysis. J Subst Abuse Treat.

[47] Lund IO, Bukten A (2015). Harm to others from substance use and abuse: the underused potential in nationwide registers. Subst Abuse.

[48] (2024). Pasienter i tverrfaglig spesialisert rusbehandling. Helsedirektoratet.

[49] Harris PA, Taylor R, Thielke R, Payne J, Gonzalez N, Conde JG (2009). Research Electronic Data Capture (REDCap)—a metadata-driven methodology and workflow process for providing translational research informatics support. J Biomed Inform.

[50] Harris PA, Taylor R, Minor BL, Elliott V, Fernandez M, O'Neal L, McLeod L, Delacqua G, Delacqua F, Kirby J, Duda SN (2019). The REDCap Consortium: building an international community of software platform partners. J Biomed Inform.

[51] Kokkevi A, Hartgers C (1995). EuropASI: European adaptation of a multidimensional assessment instrument for drug and alcohol dependence. Eur Addict Res.

[52] Lauritzen G, Ravndal E (2009). Introduction of the EuropASI in Norway: clinical and research experiences from a cost‐effectiveness study. J Subst Use.

[53] Fredheim OMS, Borchgrevink PC, Landmark T, Schjødt B, Breivik H (2008). Et nytt skjema for kartlegging av smerter [A new questionnaire for assessing pain]. Tidsskr Nor Legeforen.

[54] Cleeland C S, Ryan K M (1994). Pain assessment: global use of the Brief Pain Inventory. Ann Acad Med Singap.

[55] Klepstad P, Loge JH, Borchgrevink PC, Mendoza TR, Cleeland CS, Kaasa S (2002). The Norwegian Brief Pain Inventory questionnaire: translation and validation in cancer pain patients. J Pain Symptom Manage.

[56] Goodman LA, Corcoran C, Turner K, Yuan N, Green BL (1998). Assessing traumatic event exposure: general issues and preliminary findings for the Stressful Life Events Screening Questionnaire. J Trauma Stress.

[57] Weathers FW, Litz B, Herman D, Huska J, Keane T (1993). The PTSD Checklist (PCL): Reliability, Validity, and Diagnostic Utility.

[58] Tinkler L, Hicks S (2011). Measuring Subjective Well-Being.

[59] Berg N, Berglund F, Lund K (2018). Evaluering og testing av spørreundersøkelse om livskvalitet. Statistics Norway.

[60] Haslbeck JMB, Waldorp LJ (2020). mgm: estimating time-varying mixed graphical models in high-dimensional data. J Stat Soft.

[61] Jones PJ, Ma R, McNally RJ (2021). Bridge centrality: a network approach to understanding comorbidity. Multivariate Behav Res.

[62] Epskamp S, Borsboom D, Fried EI (2018). Estimating psychological networks and their accuracy: a tutorial paper. Behav Res Methods.

