# Supplementary material for: Chronic Pain and Posttraumatic Stress Among Patients in Substance Use Treatment: Protocol for NOR-APT, a Longitudinal Cohort Study
Source: JMIR Res Protoc. 2025 Dec 24;14:e67663. doi: 10.2196/67663 (PMC12780707; doi:10.2196/67663)
Supplement: Multimedia Appendix 1 [file resprot_v14i1e67663_app1.pdf]

Assessment of grant application submitted to the Research Council of Norway

Grant application

Project number	345355
Project title	Substance use disorder treatment: the role of comorbid chronic pain and post-traumatic stress in maintaining substance use
Project manager	Skjærvø, Ingeborg
Project owner	AKERSHUS UNIVERSITETSSYKEHUS HF
Application type	Researcher Project / Young Research Talents
Topic	Ground-breaking research
Programme/Activity	Fri prosjektstøtte
Case officer	Jostein Holmgren

Confirmation

By completing and submitting this form, I / we confirm the following (applies for the individual referee or the referee panel):

- I/We have no conflicts of interest that would prevent me/us from conducting this assessment. See Regulations on Impartiality and Confidence in the Research Council of Norway.	Yes
- I/We have read and understood both the criteria I/we have been asked to use for assessing the application and the description of the scale of marks. The scale of marks is to be applied as an absolute scale, i.e. marks are to be determined for each grant application independently and not relative to other applications that the panel/referee is assessing.	Yes
- I/We understand and accept the guidelines for assessing applications for the Research Council of Norway. See Guidelines for referees/panels who assess applications for the Research Council of Norway.	Yes
- I am/We are qualified to conduct this assessment.	Yes

Summary of marks

Criterion	Mark
Excellence – potential for advancing the state-of-the-art	6
Excellence – quality of R&D activities	6
Impact	7
Implementation	6

Criteria

Excellence – potential for advancing the state-of-the-art

.

The extent to which the proposed work is ambitious, novel, and goes beyond the state-of-the-art
• Scientific creativity and originality.
• Novelty and boldness of hypotheses or research questions.
• Potential for development of new knowledge beyond the current state-of-the-art, including significant theoretical, methodological, experimental or empirical advancement.

This is a foremost clinically oriented and driven proposal that is original and creative and that aims to better quantify the comorbidity with PTSD and chronic pain among SUD patients and understand temporal sequences issues and relevant risk (and protective) factors. The proposal addresses a very relevant clinical and societal problem that so far has been under-researched and is timely.
The proposal presents very nice and useful pilot data in Figure 1. The proposal is very well written, and clear.

As a minor weakness, the proposal is exploratory and descriptive, and lacks guidance by top-down hypotheses.

Selected mark : 6 - Excellent
The proposal successfully addresses all relevant aspects of the criterion. Only minor shortcomings are present.

Excellence – quality of R&D activities

.

The quality of the proposed R&D activities
• Quality of the research questions, hypotheses and project objectives, and the extent to which they are clearly and adequately specified.
• Credibility and appropriateness of the theoretical approach, research design and use of scientific methods. Appropriate consideration of interdisciplinary approaches.
• The extent to which appropriate consideration has been given to ethical issues, safety issues, gender dimension in research content, and use of stakeholder/user knowledge if appropriate.

The design and approach are of high quality.
Strengths of the proposal are: 1) It is a combination of large-sale quantitative research (using various registry and in part longitudinal databases including prescription databases) and qualitative research; 2) it involves collaboration with four specialty clinics, ensuring and resulting in 1500 participants/patients at the end.

Weaknesses of the proposal are: 1) It is unclear what the distribution of different subtypes of SUD among the specialty clinics is. Will it cover addiction to alcohol, opioids, benzodiazepines, heroin, etc.? 2) It is unclear whether information of previous and concurrent non-pharma treatments (and their effects) will be available and analyzed.

Selected mark : 6 - Excellent
The proposal successfully addresses all relevant aspects of the criterion. Only minor shortcomings are present.

Impact

.

Potential impact of the proposed research
• Potential for academic impact:
The extent to which the planned outputs of the project address important present and/or future scientific challenges. The extent to which the planned outputs are openly accessible to ensure reusability of the research outputs and enhance reproducibility.
• Potential for societal impact (if addressed by the applicant):
The extent to which the planned outputs of the project address UN Sustainable Development Goals or other important present and/or future societal challenges.
• The extent to which the potential impacts are clearly formulated and plausible.

Communication and exploitation
• The extent to which the appropriate open science practices are implemented as an integral part of the proposed project to ensure open sharing and wide distribution of research outputs.
• Quality and scope of communication and engagement activities with different target audiences, including relevant stakeholders/users.

The proposal makes a strong point that SUD patients are denied access to PTSD treatment in some centers. The potential impact on the organization and access criteria for SUD clinics will be huge, as well as the impact on clinical guidelines for the assessment and treatment of SUD. The project may lead to recalibrate programmes on early recognition of chronic pain and PTSD and give input to preventative programmes for SUD.

It is not explicitly mentioned here but the oxycodon scandal in the USA and the story of the Sackler family demonstrates that frequent prescription of strong analgesics in the USA by GPs, is very dangerous and can provoke the chronic pain - SUD connection. So, this project may have to deliver important messages not only for those in SUD specialty clinics but for GPs and other medical specialties and clinical psychologists as well.

Selected mark : 7 - Exceptional
The proposal addresses all relevant aspects of the criterion exceptionally well. Shortcomings are not present, or only very minor.

Implementation

.

The quality of the project manager and project group
• The extent to which the project manager has relevant expertise and experience, and demonstrated ability to perform high-quality research (as appropriate to the career stage).
• The degree of complementarity of the participants and the extent to which the project group has the necessary expertise needed to undertake the research effectively.

The quality of the project organisation and management
• Effectiveness of the project organisation, including the extent to which resources assigned to work packages are aligned with project objectives and deliverables.
• Appropriateness of the allocation of tasks, ensuring that all participants have a valid role and adequate resources in the project to fulfil that role.
• Appropriateness of the proposed management structures and governance.

The project team covers all relevant expertise. The first applicant has experience with SUD and chronic pain clinical work and research, the scientific credentials however (8 papers in PubMed) are thin. This has of course to be put into context, PhD finished 2018, and a career break for parental leave.
The other team members are more senior and will certainly be supporting and coaching. The organization of the project and breakdown in WPs seems adequate.

Selected mark : 6 - Excellent
The proposal successfully addresses all relevant aspects of the criterion. Only minor shortcomings are present.

Special points to consider

Comments to special points to consider
